# Hip Flexor Muscle Activation Across Gait Phases in Healthy Young Adults: Effects of Step Length and Cadence Adjustments at a Constant Walking Speed

**DOI:** 10.7759/cureus.84130

**Published:** 2025-05-14

**Authors:** Takumi Jiroumaru, Yutaro Hyodo, Michio Wachi, Yasumasa Oka, Takamitsu Fujikawa

**Affiliations:** 1 Department of Physical Therapy, Bukkyo University, Kyoto, JPN; 2 Rehabilitation, Kanazawa Orthopaedic and Sports Medicine Clinic, Shiga, JPN

**Keywords:** cadence, electromyography, gait, hip flexors, iliopsoas, muscle activation, step length

## Abstract

Background

Gait impairment is a critical issue in aging populations and is often characterized by a reduced step length and increased cadence. Although the iliopsoas (IL) and other hip flexors are essential for gait, the neuromuscular mechanisms underlying these compensatory changes remain unclear.

Objective

To investigate the phase-specific electromyographic (EMG) activity of the IL, sartorius (SA), rectus femoris (RF), and tensor fasciae latae (TFL) muscles during walking tasks that independently manipulate step length and cadence at a fixed walking speed.

Methods

In this exploratory study, nine healthy adult males performed the following three treadmill walking conditions at 5 km/h: (1) long step/low cadence, (2) normal gait, and (3) short step/high cadence. Surface EMG data from the four hip flexor muscles were collected and analyzed across the four gait phases. A motion-capturing system was used to assess kinematic parameters. EMG data were normalized to maximal voluntary isometric contraction (MVIC). Friedman tests with Benjamini-Hochberg false discovery rate (FDR) correction were used to compare muscle activity among the conditions.

Results

The IL activity increased significantly during the late swing phase in the long-step condition, whereas the SA and RF showed early activation in the late stance phase under the high-cadence condition. The TFL demonstrated sustained activation during the late swing phase in the high-cadence condition.

Conclusions

The present study suggests that phase-specific activation patterns in hip flexor muscles may be influenced by changes in step length and cadence, even when walking speed is held constant. The IL appears to contribute to step-length extension, while the RF and SA may play a role in swing initiation under high-cadence conditions. Sustained activation of the TFL during terminal swing may be associated with pelvic and lower limb control. Although based on a small and specific sample, these findings offer preliminary insights into neuromuscular control of gait and may serve as a useful reference for future studies, particularly those focused on age-related gait adaptations or interventions.

## Introduction

Walking ability is a key indicator of overall health in older adults and has recently gained attention as an early marker of cognitive decline, increased fall risk, and higher mortality [1-3]. Notably, changes in gait patterns often occur before limitations in activities of daily living (ADLs) and decreases in quality of life (QoL), prompting the growing recognition of gait function as the “sixth vital sign” [3]. Age-related declines in muscle strength, flexibility, and balance contribute to altered gait characteristics, such as reduced walking speed, shorter step length, increased cadence (steps per minute), and wider step width, which are directly linked to greater fall risks and reduced mobility [4-7].

Walking speed is defined as the product of the step length and cadence. While younger individuals typically increase their walking speed by lengthening their steps, older adults often compensate for their inability to do so by increasing cadence [5,8-10]. Although this strategy may help preserve dynamic stability in the context of muscle weakness, it is also associated with increased fall risk and reduced mobility in older adults [4-7]. However, the underlying neuromuscular mechanisms are not fully understood. Among the hip flexor muscles, the iliopsoas (IL) plays a central role in initiating the swing phase of the gait and controlling step length [11-13]. In addition, the IL is believed to contribute to lumbar and pelvic stability and to help maintain trunk posture [14,15]. However, the effects of specific gait modifications, particularly changes in step length and cadence, on the activation of the IL and other hip flexors remain underexplored, and their functional distinctions remain unclear.

Most previous studies have assessed electromyographic (EMG) activity in response to changes in walking speed. Studies that isolate step length and cadence as independent variables for direct comparison are limited [10,11]. However, the characteristic gait of many older adults, a combination of reduced step length and increased cadence, is distinct and clinically relevant. Understanding the neuromuscular basis of this pattern is essential for the development of rehabilitation interventions aimed at fall prevention and gait retraining.

This exploratory study aimed to quantitatively compare the EMG activity in the IL and other hip flexor muscles, including the sartorius (SA), rectus femoris (RF), and TFL, under three walking conditions at a constant speed in a small sample of healthy young adults. While the broader clinical interest lies in age-related gait impairment, we specifically selected younger participants to isolate neuromuscular activation patterns without age-related variability. As such, the findings are not generalizable to older or clinical populations and should be interpreted within the context of this narrow sample. Nonetheless, this approach provides foundational insights that may inform future studies in older populations.

## Materials and methods

Study participants

Nine healthy adult males were included in this study (mean ± SD: age, 25.6 ± 3.7 years; weight, 65.8 ± 1.9 kg; height, 176.1 ± 5.4 cm). All participants received both oral and written explanations of the study objectives and procedures. Written informed consent was obtained from each participant. The study was conducted in accordance with the ethical standards outlined in the Declaration of Helsinki and approved by the Kanazawa Orthopaedic Sports Medicine Clinic Ethics Committee (approval number: Kanazawa-OSMC-2022-008).

Experimental protocol

Participants performed three walking tasks on a treadmill (AUTORUNNER; Minato, Osaka, Japan): (1) long step length with low cadence (SL: step length = 1.0 m, cadence = 80 steps/min), (2) normal walking (N: free walking), and (3) short step length with high cadence (P: step length = 0.4 m, cadence = 190 steps/min). For the SL and P conditions, step cadence was regulated using auditory cues from a metronome. No external rhythm was applied during normal walking, allowing the participants to walk at a self-selected pace.

Each participant completed a familiarization session at least one week prior to the experimental trial to become accustomed to the walking conditions. On the day of the experiment, the participants completed a 2-minute warm-up for each task. The order of the walking conditions was randomized. Data were recorded for at least five consecutive gait cycles (approximately 10 s per trial), with a 3-minute rest period between trials. To support accurate step length control in the SL and P conditions, visual floor markers were applied to the treadmill as cues. Participants also received brief verbal reminders between trials to maintain target values.

Surface electromyography

Surface electromyography (sEMG) signals were recorded from the right-side hip flexor muscles, including the IL, RF, SA, and TFL. A 16-channel active electrode system (MQ8/16, 16-bit EMG amplifier; Kissei Comtec, Nagano, Japan) was used for data acquisition. To minimize signal cross-talk, electrodes measuring 10 × 10 mm with a narrow 10 mm inter-electrode distance were used. Placement followed the SENIAM recommendations (http://www.seniam.org/) regarding orientation along muscle fibers. Standard skin preparation, including shaving, alcohol cleansing, and degreasing, was performed to reduce impedance. In addition, raw EMG signals were visually inspected during data collection to identify and exclude traces exhibiting crosstalk artifacts. These measures ensured signal specificity and improved data validity. A reference electrode was placed over the right patella.

Electrodes were placed according to anatomical landmarks and SENIAM guidelines. To ensure accurate and consistent placement, ultrasonographic (LOGIQ P5; GE Healthcare, Chicago, USA) guidance was used for both superficial and deep muscles, including the IL, to confirm muscle boundaries and fiber orientation. All electrode placements were performed by the same experienced examiner. Inter-electrode distances were standardized at 10 mm, and electrodes were aligned parallel to muscle fibers following SENIAM recommendations. Following a previous study, electrodes for the IL were placed along the muscle fascia 3-5 cm distal to the anterior superior iliac spine (ASIS) [16]. This placement location was based on our previous validation study, which confirmed that surface EMG signals recorded from this region show minimal cross-talk from adjacent muscles.

EMG signals were sampled at 2,000 Hz using a wireless telemetry system (MQ16; Kissei Comtec, Nagano, Japan) and processed using the Kine Analyzer software (Kissei Comtec, Nagano, Japan). The signals were full-wave rectified and bandpass filtered (10-1000 Hz) for smoothing. Each gait cycle was normalized to 100%, and the root mean square (RMS) values were calculated at 5% intervals.

MVC normalization

The EMG signals were normalized using maximal voluntary isometric contraction (MVIC), which was performed in a supine position for the IL (hip flexion at 0°and knee flexion at 90°); in a seated position for the SA and RF (hip flexion at 90° and knee flexion at 90°); and lateral recumbency for the TFL (hip flexion at 45° and knee extension at 0°). Each MVIC trial lasted for five seconds, and the peak RMS value was used to normalize the walking EMG data.

Kinematic analysis

A total of 14 reflective markers were attached to the following anatomical landmarks: the bilateral ASIS, PSIS, greater trochanter, lateral femoral epicondyle, lateral malleolus, calcaneus, and the base of the fifth metatarsal. A 3D motion capture system (four cameras, 200 Hz; UM-CAT, Unimec, Tokyo, Japan) was used to record gait kinematics, and data were analyzed using the Kine Analyzer software. A second-order Butterworth low-pass filter (cutoff frequency: 8 Hz) was applied to the motion data.

The coordinate system was defined as follows: X-axis (anterior-posterior), Y-axis (mediolateral), and Z-axis (vertical). A gait cycle was defined from one right foot contact to the next. For analysis, three consecutive gait cycles were averaged for each condition (SL, N, and P). The step length was calculated as the mean distance between the right and subsequent left foot contacts.

Phase classification

Each gait cycle was divided into four phases.

Phase 1 (Early stance): From right foot contact until hip extension angular velocity reaches zero.

Phase 2 (Late stance): From the endpoint of Phase 1 until right toe-off.

Phase 3 (Early swing): From toe-off until hip flexion angular velocity reaches zero.

Phase 4 (Late swing): From the endpoint of Phase 3 until the next right foot contact.

This classification was based on previous studies examining the relationship between hip flexor muscle activity and hip joint angular velocity [17,18].

Statistical analysis

The normality of the EMG data was assessed using the Shapiro-Wilk test. Since the data were not normally distributed, the Friedman test was used to compare walking conditions. When significant differences were observed, the Benjamini-Hochberg false discovery rate (FDR) correction method was applied. Kendall’s W was calculated to assess the effect size. Statistical significance was set at p < 0.05. All statistical analyses were performed using SPSS software (version 27.0; IBM Corp., Armonk, NY, USA). In addition to p-values, 95% confidence intervals (CIs) were calculated and reported to indicate the precision of estimated effects.

## Results

Iliopsoas muscle activity

The Shapiro-Wilk test indicated that the IL EMG data did not follow a normal distribution under any of the walking conditions (p < 0.05). Accordingly, the Friedman test was used to compare the muscle activity across the three walking conditions within each gait phase (Phases 1-4). Significant differences were observed in Phase 1 (χ²(2) = 13.56, p = 0.001, W = 0.75, 95 % CI = 0.75 - 0.90), Phase 2 (χ²(2) = 8.06, p = 0.018, W = 0.45, 95 % CI = 0.33 - 0.75), and Phase 4 (χ²(2) = 18.00, p < 0.001, W = 1.00, 95 % CI = 1.00 - 1.00), while no significant differences were observed in Phase 3.

**Figure 1:**
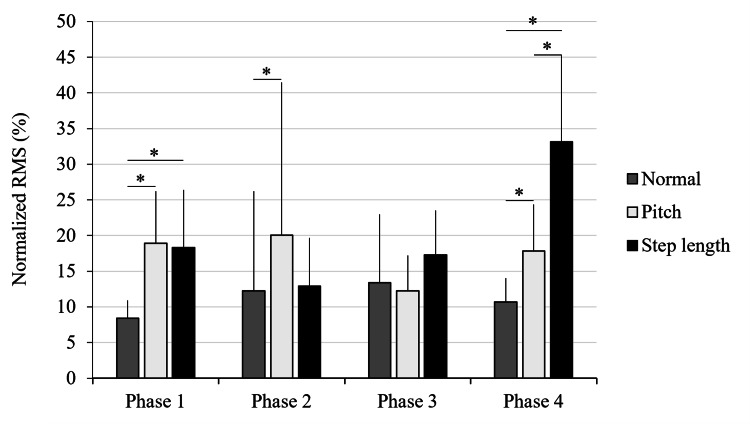
Gait phase-specific comparison of iliopsoas (IL) muscle activity across three walking conditions Mean normalized electromyographic (EMG) activity of the IL muscle during four gait phases (Phases 1–4) under three walking conditions: normal walking (N), short step/high cadence (P), and long step/low cadence (SL). Asterisks indicate significant differences between conditions as identified by the Friedman test, followed by false discovery rate (FDR) correction post-hoc analysis (*p < 0.05). Error bars represent standard deviation. Adapted from the doctoral dissertation (Ritsumeikan University, 2016; https://ritsumei.repo.nii.ac.jp/records/9445).

Post-hoc analysis using the FDR correction method revealed that in Phase 1, both the short step/high cadence (P) and long step/low cadence (SL) conditions showed significantly higher activity than normal walking (N) (p = 0.003 and p = 0.003, respectively). In Phase 2, IL activity was significantly higher in the P condition compared to the N condition (p = 0.02). In Phase 4, SL showed significantly greater activity compared to N (p < 0.001). Additionally, P exhibited significantly higher activity than N (p = 0.03), and SL showed significantly greater activity than P (p = 0.03). A gait phase-specific comparison of the IL EMG activity across the three walking conditions (SL, N, and P) is shown in Figure 1.

Sartorius muscle activity

The EMG data for the SA also failed to meet normality; thus, the Friedman test was used. Significant differences were observed in all gait phases: Phase 1 (χ²(2) = 9.31, p = 0.009, W = 0.52, 95 % CI = 0.23 - 0.93), Phase 2 (χ²(2) = 13.89, p < 0.001, W = 0.77, 95 % CI = 0.75 - 0.89), and Phase 4 (χ²(2) = 8.71, p = 0.013, W = 0.48, 95 % CI = 0.35 - 0.82) while no significant difference were observed in Phase 3.

**Figure 2:**
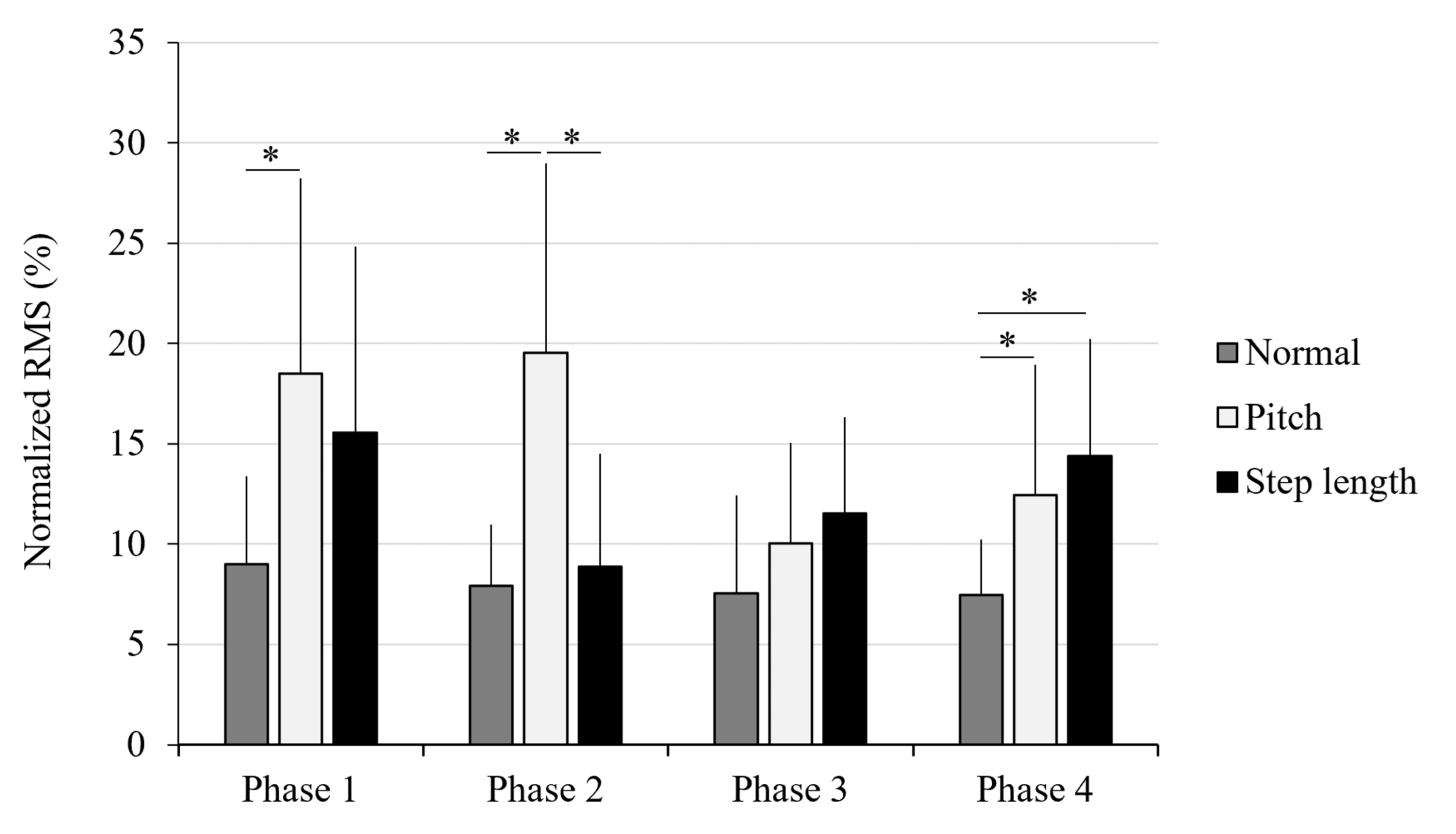
Gait phase-specific comparison of sartorius (SA) muscle activity across three walking conditions Mean normalized electromyographic (EMG) activity of the SA muscle during four gait phases (Phases 1–4) under three walking conditions: normal walking (N), short step/high cadence (P), and long step/low cadence (SL). Significant differences between conditions were identified using the Friedman test, followed by false discovery rate (FDR) correction post-hoc analysis (*p < 0.05). Error bars represent standard deviation. Adapted from the doctoral dissertation (Ritsumeikan University, 2016; https://ritsumei.repo.nii.ac.jp/records/9445).

In Phase 1, activity was significantly higher in the P condition than in the N condition (p = 0.01). In Phase 2, significant differences were observed between N and P conditions (p = 0.004) and between SL and P conditions (p = 0.004), with P showing the highest activation. In Phase 4, the SL condition showed higher activity than the N condition (p = 0.03), and the P condition also showed significantly higher activity than the N condition (p = 0.03). A gait phase-specific comparison of the SA EMG activity across the three walking conditions (SL, N, and P) is shown in Figure 2.

Rectus femoris muscle activity

Similar to other muscles, the RF EMG data did not meet the assumption of normality. The Friedman test indicated significant differences in all four gait phases: Phase 1 (χ²(2) = 12.51, p = 0.002, W = 0.70, 95 % CI = 0.60 - 0.87), Phase 2 (χ²(2) = 14.11, p = 0.001, W = 0.78, 95 % CI = 0.75 - 0.90), Phase 3 (χ²(2) = 13,56, p = 0.001, W = 0.75, 95 % CI = 0.75 - 0.90), and Phase 4 (χ²(2) = 14.11, p = 0.001, W = 0.78, 95 % CI = 0.75 - 0.94).

**Figure 3:**
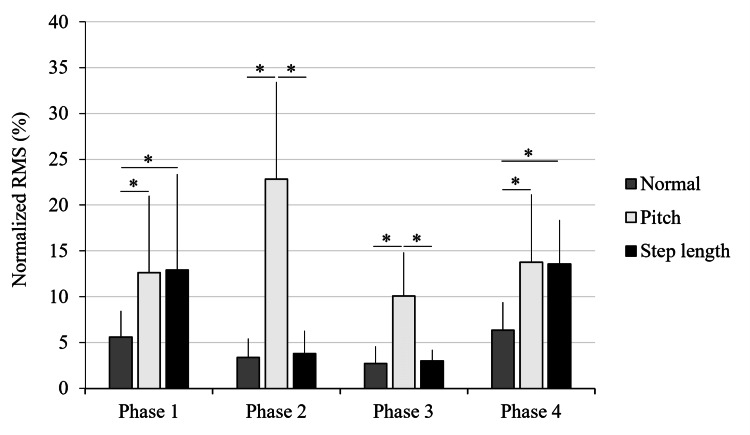
Gait phase-specific comparison of rectus femoris (RF) muscle activity across three walking conditions Mean normalized electromyographic (EMG) activity of the RF muscle during four gait phases (Phases 1–4) under three walking conditions: normal walking (N), short step/high cadence (P), and long step/low cadence (SL). Significant differences were determined using the Friedman test with false discovery rate (FDR) correction post-hoc analysis (*p < 0.05). Error bars indicate standard deviations. Adapted from the doctoral dissertation (Ritsumeikan University, 2016; https://ritsumei.repo.nii.ac.jp/records/9445).

Post-hoc analysis showed significantly higher activity in the SL and P conditions compared to N in Phase 1 (p = 0.007 and p = 0.004, respectively). In Phase 2, the P condition showed significantly greater activation than both N (p = 0.002) and SL (p = 0.005) conditions. Similar patterns were observed in Phase 3; the P condition showed significantly greater activation than both N (p = 0.003) and SL (p = 0.003) conditions. In Phase 4, both the P and SL conditions showed higher activity than N (p = 0.003 and p = 0.002, respectively). A gait phase-specific comparison of the RF EMG activity across the three walking conditions (SL, N, and P) is shown in Figure 3.

Tensor fasciae latae muscle activity

Similar to the other muscles, the TFL data were non-normally distributed. The Friedman test revealed significant differences in all phases: Phase 1 (χ²(2) = 8.40, p = 0.015, W = 0.47, 95 % CI = 0.23 - 0.82), Phase 2 (χ²(2) = 10.67, p = 0.005, W = 0.59, 95 % CI = 0.38 - 0.90), Phase 3 (χ²(2) = 13.77, p = 0.001, W = 0.77, 95 % CI = 0.56 - 1.00), and Phase 4 (χ²(2) = 17.54, p < 0.001, W = 0.97, 95 % CI = 0.94 - 1.00).

**Figure 4:**
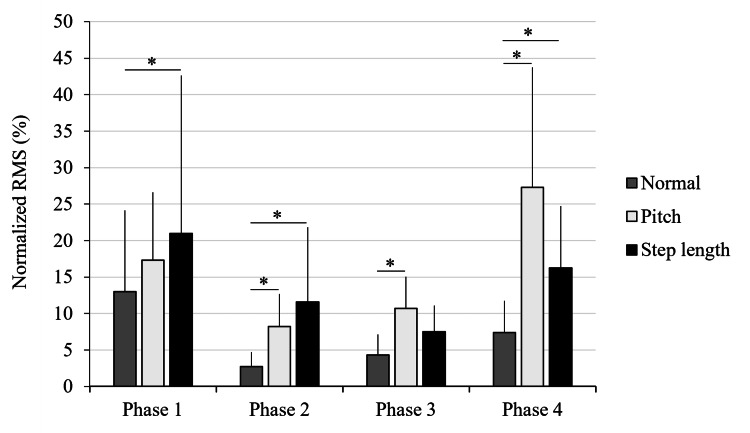
Gait phase-specific comparison of tensor fasciae latae (TFL) muscle activity across three walking conditions Mean normalized electromyographic (EMG) activity of the TFL muscle during four gait phases (Phases 1–4) under three walking conditions: normal walking (N), short step/high cadence (P), and long step/low cadence (SL). Significant differences between conditions were identified using the Friedman test, followed by false discovery rate (FDR) correction post-hoc analysis (*p < 0.05). Error bars represent standard deviation. Adapted from the doctoral dissertation (Ritsumeikan University, 2016; https://ritsumei.repo.nii.ac.jp/records/9445).

Post-hoc analysis showed significantly higher activity in the SL and P conditions compared to N in Phase 1 (p = 0.02 and p = 0.04, respectively). In Phase 2, both P and SL conditions demonstrated greater activation than the N condition (p = 0.007 and p = 0.007, respectively). In Phase 3, activity was significantly higher in the P condition compared to the N condition (p < 0.001). Similarly, in Phase 4, the P condition exhibited significantly greater activity than the N condition (p < 0.001), and the SL condition also showed significantly higher activity than the N condition (p = 0.04). A gait phase-specific comparison of TFL EMG activity across the three walking conditions (SL, N, and P) is shown in Figure 4.

Kinematic analysis of hip movement

**Figure 5:**
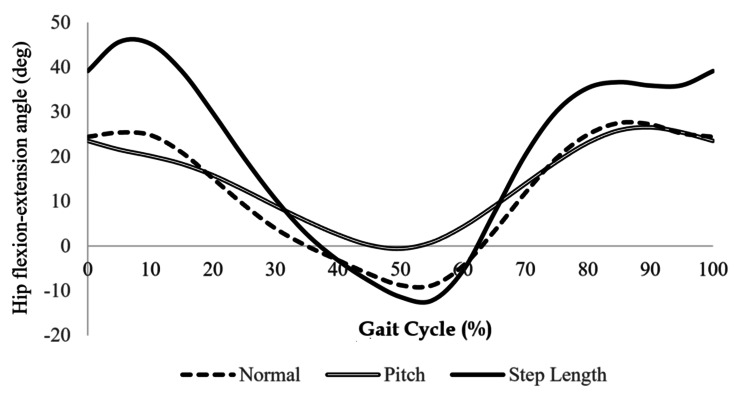
Hip flexion/extension angles during the gait cycle under different walking conditions This figure shows the changes in hip flexion angles across the gait cycle for the SL, N, and P walking conditions. The SL condition demonstrated greater hip extension during 50–60% (stance phase) and increased flexion during 70–80% and 90–100% (swing phase) of the gait cycle compared to the other conditions. Adapted from the doctoral dissertation (Ritsumeikan University, 2016; https://ritsumei.repo.nii.ac.jp/records/9445).

**Figure 6:**
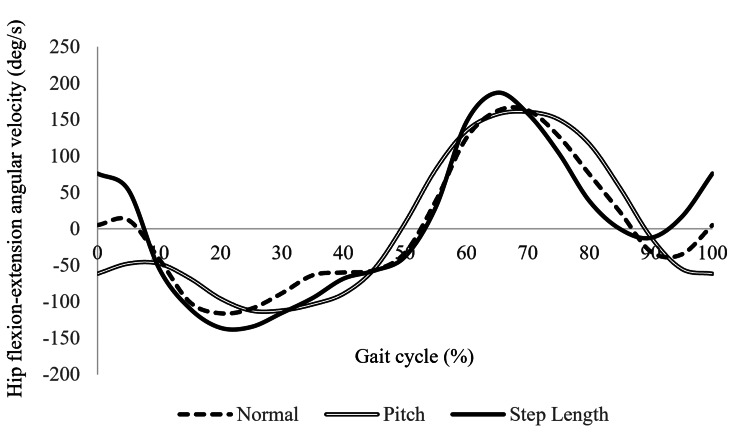
Hip angular velocities during the gait cycle under different walking conditions This figure presents the hip angular velocities for the SL, N, and P conditions. In the late swing phase (90–100% of the gait cycle), the SL condition-maintained flexion-direction velocity, while the N and P conditions transitioned toward extension. Adapted from the doctoral dissertation (Ritsumeikan University, 2016; https://ritsumei.repo.nii.ac.jp/records/9445).

Figures 5-6 show the hip flexion angles (Figure 5) and angular velocities (Figure 6) for each walking condition. During the second half of the stance phase (50-60% of the gait cycle), the SL condition exhibited a greater hip extension angle compared to the other conditions. In the early (70-80%) and late swing phases (90-100%), the SL condition showed higher flexion angles than the N or P conditions.

Regarding angular velocity, the SL condition-maintained flexion-direction movement during the late swing phase (90-100%), whereas the N and P conditions showed a shift toward extension during this period.

## Discussion

Modulation of hip flexor muscle activity in response to gait pattern alterations

This study investigated the electromyographic (EMG) activity of the IL and other hip flexor muscles - the SA, RF, and TFL -under three walking conditions in which step length and cadence were independently manipulated while maintaining a constant walking speed. The results revealed that the timing and intensity of peak muscle activation varied across gait phases and walking patterns. These findings suggest that the hip flexor muscles operate in a coordinated and phase-specific manner to accommodate changes in gait mechanics.

An increase in step length may be associated with greater IL activity during the late swing phase, while a higher cadence appears to be linked to earlier activation of the SA and RF during the late stance phase. These modulations likely represent compensatory mechanisms aimed at optimizing propulsion and maintaining stability, particularly under biomechanical constraints such as age-related muscle weakness or reduced flexibility.

Iliopsoas: a key contributor to the late swing phase and step length extension

Under the extended step length condition, the IL muscle showed significantly increased activity during Phase 4 (late swing). This phase coincides with peak hip flexion angle and angular velocity, highlighting the IL role in promoting forward propulsion and facilitating longer steps [10,16,19-21]. Importantly, the psoas major, which is the primary component of the IL, is highly susceptible to age-related atrophy [22]. This decline is associated with reduced step length, slower gait speed, impaired balance, and an increased risk [1,2,4,5,13].

Thus, the IL is not only essential for swing efficiency but also plays a pivotal role in maintaining overall gait function. These findings reinforce the clinical importance of preserving the IL function through targeted assessments and training, particularly in older adults.

Sartorius and rectus femoris: early activators supporting increased cadence

During the high cadence condition, the SA and RF demonstrated increased activity in Phase 2 (late stance), which is critical for preparing the limb for swinging. The RF likely initiates the forward motion of the leg by generating hip flexion and assisting in knee extension through their biarticular nature [17-21]. In older adults, an increased cadence is often used as a compensatory strategy to maintain walking speed despite a shortened step length [5,8-10]. Although effective in the short term, this strategy may compromise postural control and gait efficiency, thereby increasing the risk of falls and related complications.

Our findings suggest that the early activation of the SA and RF underlies the neuromuscular basis of this compensatory pattern. Accordingly, rehabilitation should aim not only to support cadence modulation but also to restore step length by strengthening the muscles involved in limb advancement.

Rectus femoris and tensor fasciae latae: muscles supporting propulsion and stability

In Phase 3 (early swing), both the RF and the TFL exhibited increased activity in the high-cadence condition. This phase demands rapid hip flexion and limb acceleration and requires significant propulsive effort. Therefore, the RF muscle is believed to be activated in response to these demands [17-21].

In Phase 4, sustained TFL activation likely aids in coordinating the pelvic-hip-limb segment to ensure a smooth transition into the stance. Given its roles in hip flexion, abduction, and internal rotation, as well as in anterior pelvic tilt and lateral balance [23,24], the TFL may contribute to both limb advancement and dynamic stability during high-cadence gait conditions. These functional contributions are especially relevant in elderly individuals, who often exhibit reduced balance and limited hip control, increasing their risk of falls. Targeted interventions focusing on the TFL function may help enhance balance and reduce instability-related gait deficits in older adults.

Coordinated activation and step length modulation across gait phases

During the long step length condition, Phase 4 was marked by increased activity of the IL, SA, and RF, suggesting their cooperative engagement in extending step length through enhanced hip flexion and angular momentum. The SA and RF likely facilitate forward swing by coordinating hip and knee joint actions [11,16,18-20].

Conversely, in the high-cadence condition, the TFL remained active throughout the late swing (Phase 4), supporting stability across the pelvis and lower limbs. This sustained activation may reflect a compensatory mechanism that supports both coordinated limb advancement and dynamic balance, particularly in populations with impaired gait control. Therefore, enhancing the TFL function through targeted neuromuscular training may hold potential clinical value in reducing fall risks.

Neuromuscular coordination and central gait control

This study highlights that adjustments in step length and cadence are accompanied by specific, phase-dependent changes in hip flexor muscle activation. The IL appears to play an important role in limb extension during the late swing phase, while the SA and RF may contribute to swing initiation in response to increased cadence. The TFL may be involved in both propulsion and postural stability, particularly during the late swing phase.

These findings support the view that gait is controlled by a complex neuromechanical system that dynamically integrates the peripheral and central signals. As proposed by Di Russo et al., reflex control parameters adapt in real time to regulate gait under varying conditions [10]. The results of the present study may offer supportive evidence for such models, suggesting that effective gait rehabilitation might benefit from considering not only muscle strength but also timing, coordination, and inter-muscle synergy.

Clinical implications for gait intervention in older adults

Age-related gait changes-including reduced step length, increased cadence, and slower walking speed-are closely linked to declines in functional independence, fall risk, and overall health outcomes [1,2,4-7,9]. Among these, shortened step length is a particularly significant early indicator of mobility impairment [3,5].

Our results suggest that preserving IL function may be important for maintaining adequate step length and walking performance. Similarly, late-phase TFL activation may support both trunk stabilization and limb advancement, suggesting its potential as a neuromuscular intervention target for improving gait control and reducing fall risk [24]. Clinical strategies incorporating selective strengthening and neuromuscular retraining of these muscles may offer an effective means to delay or reverse age-related gait decline.

Limitations

This study was conducted with a limited sample of nine healthy young adult males, selected to reduce inter-individual variability related to aging or clinical conditions. While this enabled controlled observation of muscle activity patterns, the small sample size and homogeneous demographic limit the generalizability of the findings. Given the exploratory nature of the study, the results should be interpreted with caution. A priori power analysis using G*Power (effect size f = 0.95, derived from Kendall’s W = 0.45) indicated that seven participants were sufficient to achieve a statistical power of 0.80 at an alpha level of 0.05. Our sample of nine met this requirement. Nonetheless, further research with larger and more diverse populations is necessary to confirm these findings. In particular, future studies involving older adults or clinical populations are essential to evaluate the applicability of the results to those with age-related or pathological gait impairments.

Surface EMG signals from deep muscles, such as the IL, are generally prone to signal attenuation and cross-talk from nearby superficial muscles. However, based on our previous research, we identified an optimal surface recording site for the IL with minimal cross-talk contamination. Accordingly, electrodes were placed in a region where signal specificity is relatively preserved. While ultrasonographic guidance was used to ensure accurate placement, some degree of caution remains necessary when interpreting EMG data from deep muscles. Future studies may benefit from complementary methods, such as intramuscular EMG or advanced signal decomposition techniques, to further validate and improve specificity.

The study involved only young adult males. Since gait biomechanics and neuromuscular strategies differ between sexes, due in part to pelvic morphology, joint alignment, and muscle function, the present findings may not be generalizable to female populations. Future research should investigate sex-related differences in muscle activation patterns during gait to confirm and expand on these results.

Additionally, while treadmill walking offers control over speed and cadence, it does not fully replicate the motor control strategies of natural overground gait. In particular, cadence and step length regulation may involve different sensorimotor feedback mechanisms during overground walking. Therefore, caution should be exercised when extrapolating these findings to real-world conditions. Furthermore, the use of forced gait parameters-especially the high cadence of 190 steps/min-may elicit neuromuscular responses that differ from those seen in natural aging or pathology. Although such controlled conditions help isolate specific activation patterns, they may not reflect spontaneous compensatory mechanisms.

At high cadences, soft tissue motion can introduce skin marker artifacts, potentially reducing kinematic accuracy. While markers were securely attached to anatomical landmarks, soft tissue movement cannot be eliminated. Future studies should consider using cluster marker systems or inertial measurement units (IMUs) to enhance motion capture fidelity under dynamic conditions.

Only three gait cycles per condition were analyzed for each participant. Although this decision was based on previous studies and aimed to reduce fatigue, it may not fully capture within-subject variability. Including a greater number of gait cycles in future research would improve data robustness and the representativeness of findings.

Lastly, although a three-minute rest was provided between trials to minimize fatigue, no physiological or subjective indicators (e.g., heart rate, perceived exertion) were recorded to verify recovery. Given that high-cadence walking can lead to hip flexor fatigue, especially in untrained individuals, future studies should incorporate such measures to ensure consistency and data quality.

## Conclusions

This study suggests that when walking speed is held constant, independently modifying step length and cadence leads to phase-specific changes in hip flexor muscle activity. The IL appears to contribute to step-length extension during the late swing phase, while the RF and SA are primarily active during swing initiation at high cadence. The TFL also showed increased activation during the terminal swing phase under the high-cadence condition. While these findings may reflect roles in limb advancement or dynamic stabilization, such interpretations should be approached with caution. No direct measurements of pelvic motion or kinetics were included, and EMG amplitude alone cannot fully capture complex biomechanical functions such as stabilization or propulsion.

Given these methodological limitations, the present results should be regarded as exploratory rather than conclusive. The findings provide preliminary insights into gait regulation in healthy young adults and offer a foundation for hypothesis generation. However, because the sample consisted exclusively of young males, the results are not generalizable to older or clinical populations. Therefore, any clinical implications should be interpreted cautiously. Further research involving older adults is needed to evaluate the applicability of these results in age-related gait contexts.
