# Correction: Hip Flexor Muscle Activation Across Gait Phases in Healthy Young Adults: Effects of Step Length and Cadence Adjustments at a Constant Walking Speed

**DOI:** 10.7759/cureus.c373

**Published:** 2025-11-07

**Authors:** Takumi Jiroumaru, Yutaro Hyodo, Michio Wachi, Yasumasa Oka, Takamitsu Fujikawa

**Affiliations:** 1 Department of Physical Therapy, Bukkyo University, Kyoto, JPN; 2 Rehabilitation, Kanazawa Orthopaedic and Sports Medicine Clinic, Shiga, JPN

This article has been corrected at the request of the authors to include the following:

- Primary IRB approval information was added to the ethics statement: "Primary data collection was approved by 藍野大学 (Aino University) (Aino2013-002)."
- The following attribution was added to the legends of Figures 1-6: "Adapted from the doctoral dissertation (Ritsumeikan University, 2016; https://ritsumei.repo.nii.ac.jp/records/9445).”

